# The Pathway from Gene Therapy to Genome Editing: A Nightmare or Dream

**DOI:** 10.22088/IJMCM.BUMS.8.2.69

**Published:** 2019-08-25

**Authors:** Mandana Hasanzad, Bagher Larijani

**Affiliations:** 1 *Medical Genomics Research Center, Tehran Medical Sciences, Islamic Azad University, Tehran, Iran. *; 2 *Personalized Medicine Research Center, Endocrinology and Metabolism Clinical Sciences Institute, Tehran University of Medical Sciences, Tehran, Iran. *; 3 *Endocrinology and Metabolism Research Center, Endocrinology and Metabolism Clinical Sciences Institute, Tehran University of Medical Sciences, Tehran, Iran.*

It is now 52 years since Marshall Nirenberg Nobel prize winner of "breaking the genetic code” mentioned to the reality of “genetic surgery” in science in 1967 (1).

Gene therapy is now revolutionizing health sciences and pharmaceutical industry. The gene therapy approach has largely been improved by the development of new methods in non-viral and viral gene transfer vectors. Promising gene therapy strategies against monogenic disease, such as inherited immune disorders, hemophilia, sickle cell anemia are capable to restore missing gene function by viral transfer. No "perfect vector" has been introduced that has the potential to treat every disorder although a “platform” vector has been suggested (2). An appropriate vector is the one that target the right cell, turn on the corresponding gene, and avoid side effect of gene transfer (3). As the viral vectors only mediate gene addition, new powerful genetic therapeutic technology, genome editing, has been emerged. In genome editing besides gene addition, two other approach “gene correction” and “gene ablation“ have been used (4). Genome editing in eukaryotic cells needs sequences specific nucleases. Gradual progress in transcri-ptional activator-like effector nucleases (TALEN) and clustered regularly interspaced short palindromic repeats (CRISPR) systems providing genome editing as a potential method in terms of efficacy, safety, and specificity (5).

It has now been 30 years since the first authorized gene transfer trial was launched at the NIH and now the field of gene therapy appears to be fulfilling its early potential (6). There were 2805 gene therapy clinical trials in 38 countries until the last update at August 2018. Most of the gene therapy clinical trials have targeted cancer (66%) and monogenic diseases (11.4%) (7). Kymriah^TM^ and Yescarta^TM^ are the first two gene therapy marketed products which can be applied as e*x vivo *gene therapy treatments (8, 9). Luxturna^TM^ is an *in vivo* treatment for PE65 gene mutation‐associated retinal dystrophy (10). The unit costs of gene therapy products are among the most expensive of European Medicines Agency (EMA) and U.S. Food and Drug Administration (FDA) approved products. The annual cost of gene therapies is roughly similar to other therapies such as enzyme replacement therapy (ERT) and precision molecular therapies with annualized cost between $250,000-$500,000 per year. For instance, one run cost for Kymriah^TM^, the first gene therapy FDA approved for B cell acute lymphoblastic leukemia, is $475,000 with eight extra years of life beyond what conventional treatment by almost $330,000 (8, 11, 12). Nevertheless, gene therapies market may not attain the expected return on their investment. Hopefully, in the upcoming years the same thing “dropping the cost of sequencing human genome” as seen in the era of personalized medicine, will occur for gene therapy too. We expect major step forward for gene therapy in terms of technique and price (13). As a result, a newborn with inherited genetic disorder may be diagnosed by high-throughput methods such as next generation sequencing (NGS), and then an appropriate gene modification approach will replace the deficient gene by the correct one.

Gene therapy development is not easier than drug development and harbor several obstacles, including an appropriate gene delivery system and clinical trial issues. One of the major advantages of gene therapy is “once- and-done” treatments and sustainability during life time, but it may be considered as a disadvantage because there is no way to turn gene expression off. After a long journey from bench to bedside, scientists, researchers and clinicians are visiting the fruits of their efforts in the gene therapy field in the market for leukemia and inherited forms of blindness and bringing new hope for the patients who suffer from their disease and experienced no treatment option for better quality of life. In the case of genome editing, we should consider ethical and safety concerns as it presents exciting opportunities for dealing with a number of diseases. The journey is not over yet, and this trend is expected to continue as the clinical vision develops and the technical capacity improves.

## Conflict of interest

Authors declare no conflict of interest.
